# Determination of corneal phase and group refractive indices *in situ* and dispersion *ex vivo* using a hybrid spectral-domain OCT and confocal scanning system

**DOI:** 10.1117/1.JBO.31.8.086003

**Published:** 2026-08-08

**Authors:** Jianli Ren, Xikai Wei, Cheng Lu, Qingyun Li, Kaixi Zheng, Dexi Zhu

**Affiliations:** a Eye Hospital, Wenzhou Medical University, Zhejiang Key Laboratory of Ophthalmic Drug Discovery and Medical Device Research, Wenzhou, China; b Eye Hospital, Wenzhou Medical University, National Clinical Research Center for Ocular Diseases, Wenzhou, China; c Zhejiang Lab for Regenerative Medicine, Vision and Brain Health, Wenzhou, China

**Keywords:** optical coherence tomography, confocal scanning, corneal refractive index, dispersion coefficients

## Abstract

**Significance:**

Precise determination of corneal refractive indices is essential for accurate refractive power calculations, personalized refractive surgery planning, and glaucoma management. However, current clinical methods fail to distinguish between the group refractive index ($n_{g}$) for thickness quantification and the phase refractive index ($n_{p}$) for optical power calculations, often relying on population-averaged constants that mask individual heterogeneity.

**Aim:**

We aim to develop a multimodal measurement system integrating spectral-domain optical coherence tomography (SD-OCT) and confocal scanning for the precise extraction of the $n_{g}$, $n_{p}$, and dispersion coefficients of corneal tissues.

**Approach:**

A dual-modality platform sharing an 860 nm source was constructed to jointly capture the optical path length (OPL) and confocal distance. A two-step progressive workflow was implemented: first, the $n_{g}$ and thickness were independently determined via the OPL method to serve as target ground truths for extracting tissue-specific dispersion through iterative optimization; second, these quantified priors were utilized to jointly decouple the *in situ* $n_{g}$, $n_{p}$, and thickness of intact corneas.

**Results:**

System validation using optical window plates yielded a relative error of only 0.01% to 0.09% for $n_{g}$ and 0.17% to 5.74% for dispersion coefficients. For biological tissues, we report the first direct experimental measurement of phase dispersion for human lenticules ($-0.014468\pm 0.004060\;\mu m^{-1}$) and porcine sections ($-0.010223\pm 0.002811\;\mu m^{-1}$), where the human data showed high consistency with the theoretical Cauchy model ($-0.014186\;\mu m^{-1}$). Utilizing these priors, the system successfully decoupled the *in situ* parameters of whole porcine eyes, yielding $n_{g}$ of 1.3845±0.0006 and an $n_{p}$ of 1.3757±0.0006.

**Conclusions:**

This technique successfully resolves the $n_{g}$ and $n_{p}$ decoupling challenge in corneal tissues, overcoming the limitations of relying on a single equivalent refractive index. By utilizing an empirically measured corneal dispersion coefficient as a mathematical constraint, the system enables the nondestructive decoupling of physical thickness, as well as phase and group refractive indices *in situ*. This breakthrough provides essential physical parameters to enhance clinical pachymetry precision and optimize personalized refractive surgery protocols.

## 1 Introduction

As the primary refractive element of the eye, the cornea accounts for approximately two-thirds of the total ocular refractive power.^1^^,^^2^ Precise determination of the corneal refractive index (RI)—the fundamental parameter governing optical refraction—is critical in clinical ophthalmology and optometry for accurate surgical planning and refractive correction.^3^^–^^7^ Modern customized refractive surgeries (e.g., LASIK, SMILE), achieving a targeted precision of ≤±0.25 diopters (D) is highly desired, whereas residual refractive errors exceeding ±0.50 D are universally considered clinically significant as they directly degrade uncorrected visual acuity.^8^^,^^9^ Based on paraxial optics, the calculation of corneal power depends primarily on its anterior curvature and refractive index. Consequently, theoretical models indicate that a minor deviation of just 0.004 to 0.006 in the assumed RI can introduce a clinically significant calculation error of 0.5 to 0.8 D.^7^^,^^10^ In clinical practice, corneal assessment has evolved from conventional keratometry to advanced three-dimensional tomography, such as Scheimpflug imaging (e.g., Pentacam).^11^^–^^13^ However, a systematic limitation persists across these commercial modalities: although they provide high-precision characterization of corneal geometry (i.e., curvature and elevation), they lack the capability to directly quantify the tissue’s intrinsic optical properties. Consequently, whether employing paraxial Gaussian optics or advanced ray-tracing algorithms, the calculation of refractive power invariably relies on assumed, population-averaged refractive indices—typically the fictitious keratometric index of 1.3375 or a constant stromal index of 1.376.^14^^–^^16^

Precise quantification of central corneal thickness (CCT) is indispensable for preoperative screening in refractive surgery and the diagnosis of corneal ectatic disorders.^17^^–^^20^ CCT also serves as a critical calibration factor in the assessment of intraocular pressure, which plays a pivotal role in glaucoma management.^21^^,^^22^ In current clinical practice, pachymetry is dominated by noncontact optical biometers, specifically those employing swept-source optical coherence tomography (e.g., IOLMaster 700) and optical low-coherence reflectometry (e.g., Lenstar LS 900).^23^^–^^25^ Fundamentally, these devices operate on the principle of low-coherence interferometry (LCI) to detect the time delay of backscattered light, thereby quantifying the optical path length (OPL). To retrieve physical thickness, the measured OPL is scaled by the tissue’s group refractive index.^26^^,^^27^ However, a systematic limitation exists across these modalities: commercial algorithms invariably adopt a constant, population-averaged group refractive index (e.g., 1.376) to perform this conversion.^26^

Fundamentally, a rigorous distinction must be drawn between the group refractive index ($n_{g}$) and the phase refractive index ($n_{p}$). The phase refractive index governs light refraction at interfaces according to Snell’s law,^28^ rendering it the indispensable variable for precise corneal refractive power calculations and wavefront aberration analysis.^16^^,^^29^ On the contrary, the group refractive index dictates the propagation speed of the optical wave packet^28^ and is used as the critical scaling factor in LCI to transform measured time delays into physical thickness.^30^^,^^31^ These two indices are intrinsically coupled through the chromatic dispersion of the tissue.^32^^,^^33^ Despite this established physical relationship, current clinical protocols conventionally conflate these parameters or rely on empirical constants, thereby systematically neglecting the significant discrepancies induced by the chromatic dispersion term.

Various techniques have been established to measure the refractive indices of biological tissues and transparent materials. For the determination of $n_{p}$, total internal reflection (TIR) and confocal scanning are the most prominent techniques.^34^^–^^38^ The TIR method relies on Snell’s law to derive the refractive index by precisely detecting the critical angle at the prism-sample interface. Confocal scanning estimates $n_{p}$ by mechanically translating the sample to locate the signal peaks corresponding to the anterior and posterior surfaces. By analyzing the optical displacement of these peaks using a geometric model based on Snell’s law, the phase refractive index can be calculated. Regarding the measurement of $n_{g}$, the OPL method utilizing optical coherence tomography (OCT) is widely adopted.^39^^–^^41^ In this configuration, a stationary mirror is typically placed behind the sample to serve as a fixed distal reference. The insertion of the sample introduces an additional optical delay, causing the mirror’s signal to shift in the depth profile compared with the reference state. Consequently, the group refractive index is obtained by dividing this measured OPL by the sample’s physical thickness.

The conventional techniques mentioned above are typically restricted to measuring only a single refractive index type (either phase or group). To address this limitation, hybrid measurement modalities have been employed to simultaneously quantify both refractive indices. For instance, Yao et al. demonstrated a hybrid system combining confocal scanning with Fourier domain OCT to characterize layered polymer structures.^42^ Although this dual-modal approach yields two independent measurements, confocal distance and OPL, the simultaneous retrieval of $n_{p}$ and $n_{g}$ remains mathematically under-determined. Consequently, the solution relies on prior knowledge of material dispersion properties, which were explicitly provided by the manufacturer in their study. To mitigate this dependence for unknown materials, Maruyama et al. proposed an empirically derived dispersion approximation.^43^ By statistically analyzing the properties of a vast array of standard optical materials, they established a power–law relationship between dispersion and the phase refractive index, to effectively reduce the number of variables to enable simultaneous measurement. However, as these empirical coefficients were calibrated strictly using industrial glasses and polymers, their applicability to biological tissues, which exhibit complex and heterogeneous dispersion properties, remains questionable.

Alternatively, direct measurement of the material’s chromatic dispersion offers a strategy to bypass reliance on empirical approximations. Kim et al. employed confocal scanning at discrete wavelengths to experimentally extract the frequency derivative of the phase refractive index.^44^ To circumvent the tedious manual switching of light sources required by discrete methods, Francis et al. introduced a spectral-domain low-coherence interferometry technique.^45^ By spectrally dispersing a supercontinuum broadband source across 3648 detection channels, this method achieved single-scan resolution of focal shifts over a broad wavelength range (600 to 1000 nm). However, a fundamental trade-off exists: such high spectral resolution necessitates high-power illumination to maintain an adequate signal-to-noise ratio for each wavelength. Consequently, the optical power levels required often exceed the maximum permissible exposure limits for *in vivo* ocular applications.^46^

To circumvent the complexities of multiwavelength setups, alternative approaches have relied on numerical estimation using empirical dispersion models. Most notably, Atchison and Smith^47^ systematically modeled the chromatic dispersions of human ocular media utilizing Cauchy’s equations. Building upon this theoretical baseline, Tian et al.^48^ elegantly employed the analytical derivative of Cauchy’s equation ($dn_{p}/d\lambda$) alongside experimentally measured group indices to estimate the phase index of the human cornea. Although these Cauchy-equation-based approaches provide a mathematically accessible framework, they possess a fundamental limitation: they rely entirely on historical, population-averaged dispersion coefficients. Consequently, these empirical models represent a generalized "standard" cornea, failing to account for individual biological heterogeneity. More importantly, static empirical formulas are inherently blind to the dynamic physiological state of the specific tissue under investigation, such as varying hydration levels during surgery or ultrastructural alterations in subclinical ectasia. Therefore, rather than relying on generalized mathematical assumptions, an approach capable of extracting the individualized dispersion properties through sample-specific data inversion is strictly required.

In this work, we present a multimodal system integrating spectral-domain optical coherence tomography (SD-OCT) and a confocal scanning unit to bridge this gap. A reference-based optimization strategy was employed to extract the dispersion of the tested sample. The reliability of this method was first validated using three optical glasses. Subsequently, we quantitatively determined the specific dispersion coefficients of *ex vivo* porcine corneal sections and human corneal lenticules through direct experimental measurement for the first time. Finally, utilizing the determined dispersion priors, we achieved the simultaneous *in situ* measurement of group and phase refractive indices in whole porcine eyes. The results demonstrate the system’s potential for noninvasive, *in vivo* clinical applications, such as improving ophthalmic diagnosis and refractive surgery planning.

## 2 Methods

### 2.1 Experimental Setup

**Fig. 1:**
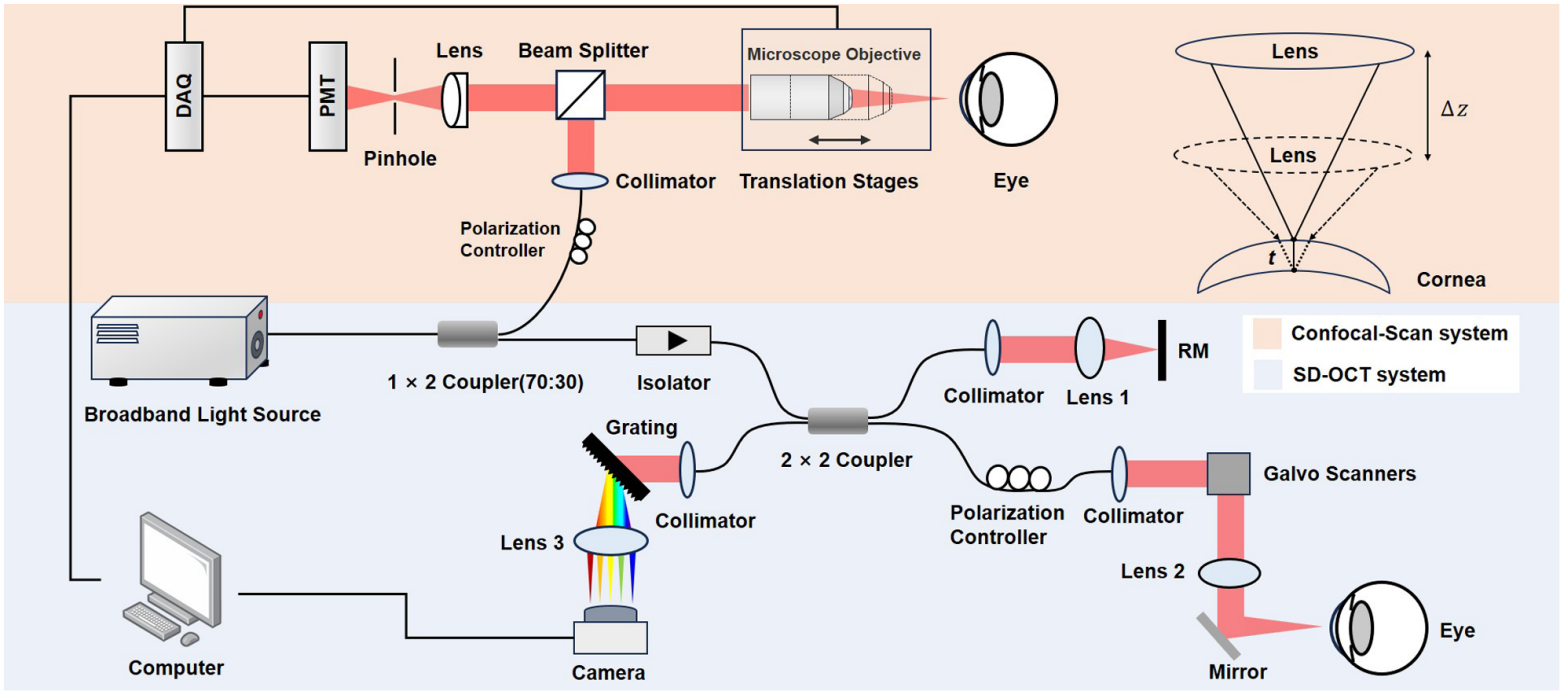
Schematic diagram of the integrated multimodal measurement system.

A schematic diagram of the experimental setup is illustrated in Fig. 1. The multimodal measurement system comprises an SD-OCT system and a confocal scanning system. A broadband superluminescent diode (BLM2-D-860-G-5, Superlum, Carrigtohill, Ireland) with a central wavelength of 860 nm and a bandwidth of 70 nm serves as a common light source for both systems. Initially, the light from the broadband source is split by a 1×2 fiber coupler, directing 70% of the power to the SD-OCT system and the remaining 30% to the confocal system.

The SD-OCT module employs a custom-built fiber-optic Michelson interferometer. The 70% broadband probing light is directed through an optical isolator to a 2×2 fiber coupler. The sample arm consists of a polarization controller, a collimator, galvanometer scanners for transverse scanning, and a focusing lens (Lens 2) to illuminate the tissue (Fig. 1). The reference arm utilizes a corresponding collimator, a focusing lens (Lens 1), and a reference mirror. The interference signal generated at the coupler is routed to a high-resolution spectrometer, comprising a collimator, a transmission diffraction grating (HD1200 1/mm, Wasatch Photonics, Morrisville, North Carolina, United States), Lens 3, and a high-speed linear array camera (EM4CL2014-BA9, E2V). Data acquisition via a frame grabber (NI PCIe-1433, National Instruments, Austin, Texas, United States) is strictly hardware-synchronized with the galvanometer. Operating at a 70 kHz A-scan rate, the system achieves an imaging depth of 2.75 mm and an axial resolution of 4.66  μm in air. B-scans are reconstructed via standard fast Fourier transform processing.

In the confocal system, the light beam is collimated and subsequently directed onto a beamsplitter (BSN11R, Thorlabs, Newton, New Jersey, United States). The reflected beam is then focused onto the sample through a 10× microscope objective (PlanC N 10×/0.25, NA=0.25, Olympus, Tokyo, Japan). A precision motorized linear translation stage (MTS50/M-Z8, Thorlabs) is mounted beneath the objective to facilitate axial translation of the focal point. This stage features tunable velocity, programmable acceleration profiles, and a minimum trigger step size of 1  μm. For the measurements in this study, the stage operates at a scanning velocity of 0.5  mm/s and an acceleration of $1.5\;\mathrm{mm}/s^{2}$ to ensure stable motion. The reflected light from the sample is collected by the same objective lens and propagates back through the beamsplitter. The returning light is then focused by an achromatic doublet lens (AC254-030-B-ML, Thorlabs), and transmitted through a pinhole (P10K, Thorlabs) to effectively block stray light originating from out-of-focus planes, thereby enabling optical sectioning capability. The optical signal is finally detected by a high-sensitivity photomultiplier tube (H10723-20, Hamamatsu, Hamamatsu City, Japan), and digitized by a data acquisition card (DAQ, PCIe-6351, National Instruments) operating at a sampling rate of 1 kHz. The movement of the translation stage and the data acquisition are synchronized by the DAQ. By recording the voltage signal at each scanning location along the axial direction, the depth information of the sample can be reconstructed. Using a 10  μm pinhole, the system yields a theoretical lateral resolution of ∼1.27  μm and an axial resolution of ∼23.8  μm.

To ensure exact spatial co-registration between the physically separated SD-OCT and confocal modules, specific alignment protocols were implemented. For *in vitro* measurements, both modules were strictly anchored to the geometric center of the regular circular samples (porcine slices and human lenticules). In the SD-OCT module, the geometric center was identified using orthogonal cross-scans. Subsequently, a customized infrared viewing card with a matching concentric hollow aperture was utilized to lock the confocal beam onto the exact same geometric center. For *in situ* whole eye measurements, the corneal apex was utilized as the absolute anatomical landmark. The real-time OCT preview ensured orthogonal incidence at the apex, and the sample was then fine-tuned in the confocal module using the IR card to illuminate the exact same apical region.

### 2.2 Porcine Cornea Preparation

Fresh porcine eyeballs were obtained from a local slaughterhouse and transported to the laboratory within 12 h of enucleation. Prior to experimentation, all eyes were inspected, and those exhibiting corneal opacity or mechanical damage were excluded to ensure optimal optical quality. A total of nine porcine eyes were included in the study. Five eyes were utilized for the dispersion optimization experiments, where a central corneal section with a diameter of 8 mm was excised from each eye using a custom-made annular cutter. During the data acquisition of these excised sections, phosphate-buffered saline (PBS) was periodically applied to the sample surfaces to maintain physiological hydration. The remaining four eyes were maintained intact for the *in situ* whole-eye measurements.

### 2.3 Human Corneal Lenticule Preparation

This study was conducted in accordance with the tenets of the Declaration of Helsinki and was approved by the Ethics Committee of the Eye Hospital of Wenzhou Medical University. The requirement for informed consent was waived by the Ethics Committee. Human corneal stromal lenticules were obtained from patients undergoing small incision lenticule extraction (SMILE) refractive surgery at the Eye Hospital of Wenzhou Medical University. The surgeries were performed using a VisuMax femtosecond laser system (Carl Zeiss Meditec AG, Jena, Germany). A total of three corneal lenticule samples were collected from three male subjects (aged 18, 19, and 20 years, respectively). Each extracted lenticule had a uniform diameter of 7.0 mm. Immediately following surgical excision, the samples were immersed in PBS and stored at 4°C to preserve physiological hydration. To minimize post-mortem tissue degradation and swelling-induced variations in optical properties, all measurements were completed within 12 h of extraction.

### 2.4 Principles

Based on the low-coherence interferometry principle of OCT, the depth information derived from the interference signal corresponds to the OPL of the sample rather than its physical thickness. The OPL, denoted as ΔD, measured as the axial separation between the anterior and posterior surfaces in the SD-OCT image, is defined as the product of the group refractive index ($n_{g}$) and the physical thickness (t):^41^^,^^27^

$$\Delta D=n_{g}\times t.$$ (1)

In the confocal scanning system, the measured confocal distance (ΔZ) differs from the actual physical thickness due to the refractive index mismatch between the sample and the surrounding medium. According to geometrical optics, the relationship between the physical thickness (t) and the phase refractive index ($n_{p}$) is^38^

$$\Delta z=t\times \frac{\sqrt{n_{\text{air}}^{2}-{\mathrm{NA}}^{2}}}{\sqrt{n_{p}^{2}-{\mathrm{NA}}^{2}}},$$ (2)

where NA is the numerical aperture of the objective lens in the confocal system, which is 0.25 in our system. $n_{\text{air}}$ is the refractive index of air, taken as 1 in this work.

Furthermore, the group refractive index ($n_{g}$) and the phase refractive index ($n_{p}$) are intrinsically linked through the material’s chromatic dispersion properties. At a central wavelength $\lambda_{c}$, this dependence is expressed as^28^

$$n_{g}=n_{p}-\lambda_{c}{\left(\frac{dn_{p}}{d\lambda }\right)}_{\lambda_{c}},$$ (3)

where $dn_{p}/d\lambda$ denotes the chromatic dispersion coefficient at λc (hereafter denoted as $\eta_{d}$), which typically assumes a negative value.

### 2.5 Measurement Procedure and Data Processing

**Fig. 2:**
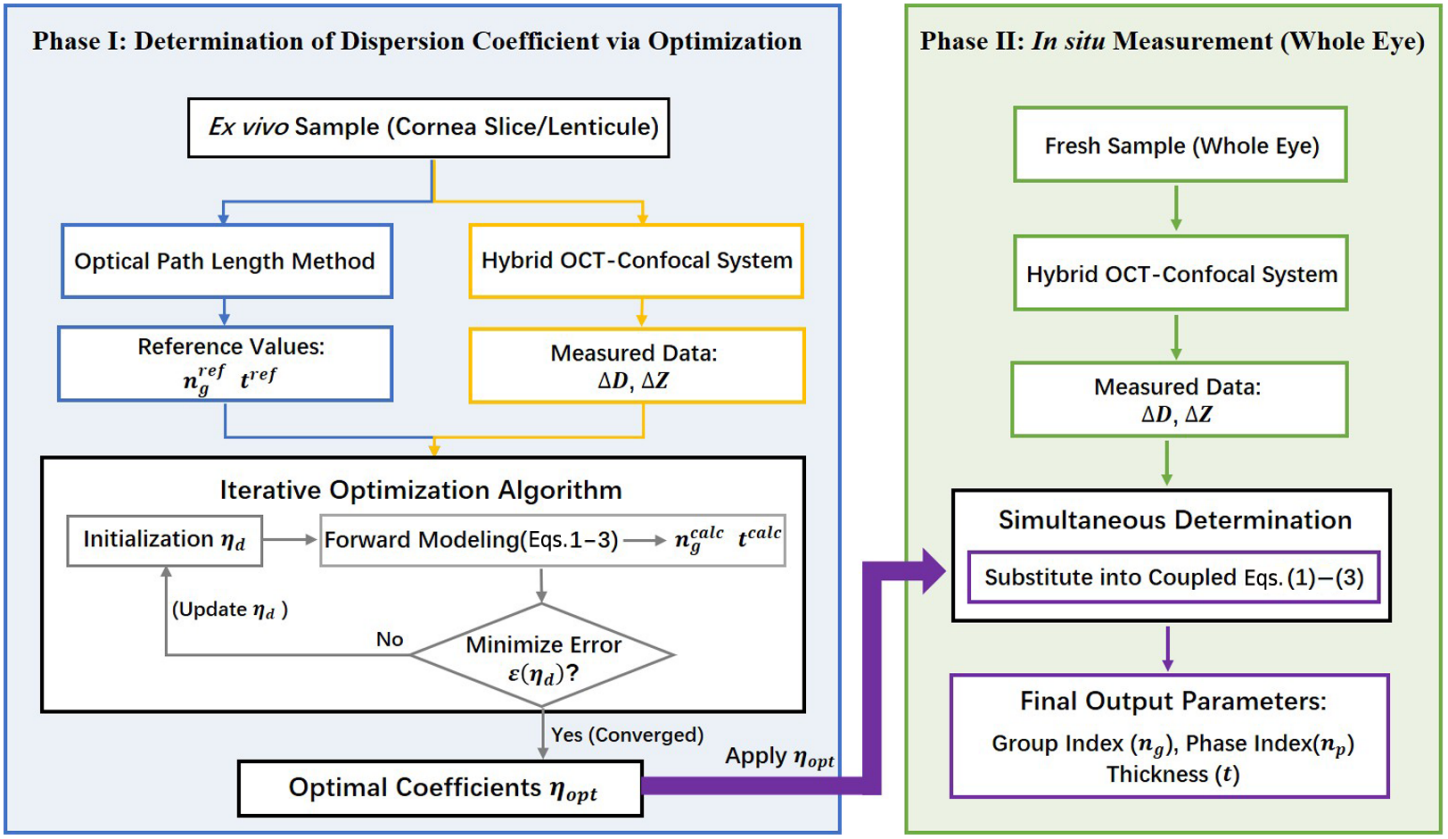
Flowchart of the measurement and data processing framework. In phase I, the error function $\varepsilon (\eta_{d})$ is minimized by comparing the forward-modeled values with the target values ($n_{g}^{\mathrm{mea}}$, $t^{\mathrm{mea}}$) acquired from the OPL method. Phase II utilizes the optimized coefficient $\eta_{\mathrm{opt}}$ to simultaneously decouple $n_{g}$, $n_{p}$, and t in whole eyes.

The measurement and data processing framework is divided into two distinct phases, as illustrated in the flowchart in Fig. 2. Phase I focuses on the determination of the specific dispersion coefficient using *ex vivo* corneal sections, whereas phase II applies this optimized parameter to achieve *in situ* measurement for fresh samples.

#### 2.5.1 Phase I: determination of dispersion coefficient via optimization

**Fig. 3:**
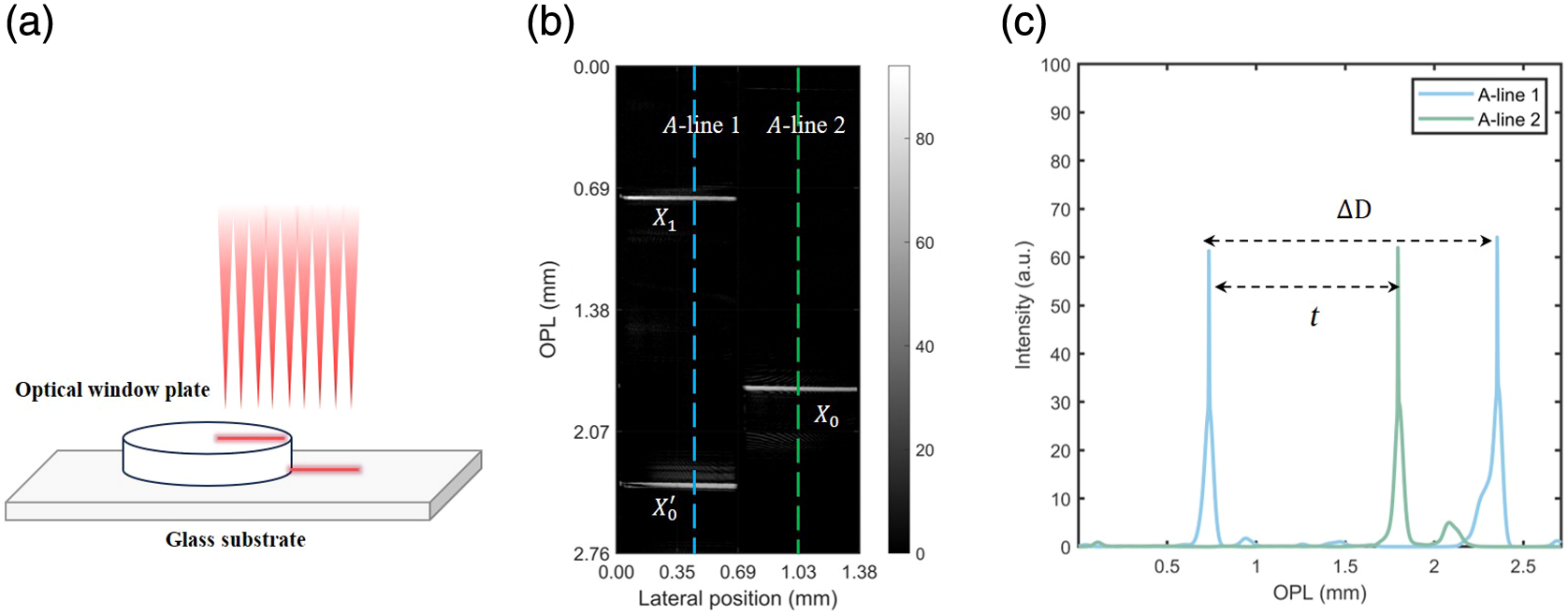
Measurement of group refractive index and physical thickness using the OPL method. (a) Schematic diagram of the tested optical window plate sample placed on a glass substrate. (b) SD-OCT B-scan image. The vertical dashed lines indicate the selected A-lines. (c) Intensity profiles along the two labeled A-lines. These measurements were obtained using an N-BK7 window with a thickness of ∼1  mm. $X_{1}$, the OCT depth of the front surface of the sample. $X_{0}^{\prime }$, the OCT depth of the front surface of the substrate beneath the sample. $X_{0}$, the OCT depth of the front surface of the bare substrate. ΔD, optical path length. t, sample thickness.

**Fig. 4:**
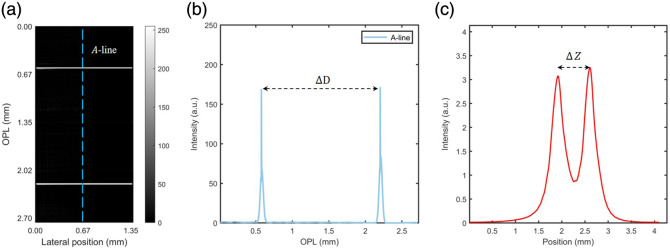
Measurement of optical path length and confocal distance of an optical window plate. (a) SD-OCT B-scan image. The vertical dashed line indicates the selected A-line. (b) Intensity profile along the labeled A-line. (c) Confocal intensity profile. These measurements were obtained using an N-BK7 window with a thickness of ∼1  mm. ΔD, optical path length. ΔZ, confocal distance.

The dispersion determination procedure comprises the following sequential steps:

(1) Acquisition of target values: To establish the “ground truth” for the optimization algorithm, the measured group refractive index ($n_{g}^{\mathrm{mea}}$) and physical thickness ($t^{\mathrm{mea}}$) of the *ex vivo* porcine corneal sections and SMILE-derived human corneal lenticules were precisely determined using the OPL method.^41^ For system verification in the experiment, three types of optical window plates, N-BK7, calcium fluoride (${\mathrm{CaF}}_{2}$), and fused silica, were adopted as standard reference samples because the refractive index and dispersion coefficient of these glass materials are known. As depicted in Fig. 3(a), the sample was placed on a glass substrate, and a line-scanning protocol covering the sample region and the bare substrate region was executed by the SD-OCT system. Specifically, the transverse scanning range of the B-scan was set to 1.38 mm (consisting of 1024 A-lines) for optical window plates, and it was adjusted to 6.29 mm (consisting of 2048 A-lines) for corneal tissues. The presence of the sample induces an optical path delay, causing the position of the substrate’s front surface to shift from $X_{0}$ (without sample) to $X_{0}^{\prime }$ (with sample) in the OCT image, as shown in Fig. 3(b). In calculating the optical path difference, the interface signals ($X_{1}$, $X_{0}^{\prime }$, and $X_{0}$) were averaged over a region spanning 30 adjacent pixels (A-lines) to ensure data robustness and mitigate localized noise. Based on the positions of the front surfaces of both the sample ($X_{1}$) and the substrate extracted from the intensity profile [Fig. 3(c)], the target values were calculated using the following relations:
  $$t=X_{1}-X_{0},$$ (4)
  $$n_{g}=\frac{\left(X_{1}-{X_{0}}^{\prime }\right)}{t}=\frac{\left(X_{1}-{X_{0}}^{\prime }\right)}{\left(X_{1}-X_{0}\right)}.$$ (5)
(2) Hybrid system measurement: Subsequently, the same sample was measured using the multimodal system. As shown in Fig. 4, B-scan images were acquired through three repeated measurements at the same position for each sample during the OCT acquisition. The ΔD was extracted from each OCT A-lines and was subsequently averaged to obtain the final mean ΔD. To determine the ΔZ, smoothing and low-pass filtering were first applied to the raw confocal intensity profile and then followed by peak alignment and averaging across these three repeated measurements. Finally, ΔZ was obtained by extracting the spatial coordinates of the maximum intensity peaks from the consolidated profile.
(3) Iterative optimization algorithm: With the target values ($n_{g}^{\mathrm{mea}}$, $t^{\mathrm{mea}}$) and measurement data (ΔD, ΔZ), a numerical optimization was performed to identify the optimal dispersion coefficient. The procedure is as follows:
  Step i: Initialization. To ensure convergence and prevent local minima, the initial search boundaries for dispersion coefficients ($\eta_{d}$) were physically constrained: bounds for optical window plates were guided by their nominal values, whereas bounds for corneal tissues were referenced to the dispersion of pure water due to their high hydration.
  Step ii: Forward modeling. For a given $\eta_{d}$, the theoretical group refractive index ($n_{g}^{\mathrm{calc}}$) and thickness ($t^{\mathrm{calc}}$) were computed by solving the coupled Eqs. (1)–(3).
  Step iii: Error evaluation. A composite error function $\varepsilon (\eta_{d})$ was constructed to quantify the deviation between the calculated values and the target values based on a weighted least-squares principle:
    $$\varepsilon (\eta_{d})=\omega_{1}{\left[\frac{\left(n_{g}^{\mathrm{calc}}(\eta_{d})-n_{g}^{\mathrm{mea}}\right)}{n_{g}^{\mathrm{mea}}}\right]}^{2}+\omega_{2}{\left[\frac{\left(t^{\mathrm{calc}}(\eta_{d})-t^{\mathrm{mea}}\right)}{t^{\mathrm{mea}}}\right]}^{2},$$ (6)
    where $\omega_{1}$ and $\omega_{2}$ represent weighting coefficients (set to $\omega_{1}=\omega_{2}=0.5$).
  Step iv: Optimization. The Quasi-Newton algorithm was employed to iteratively minimize $\varepsilon (\eta_{d})$, thereby converging to the optimal dispersion coefficient $\eta_{\mathrm{opt}}$.

#### 2.5.2 Phase II: simultaneous retrieval of phase and group refractive indices

Once the dispersion coefficient $\eta_{\mathrm{opt}}$ was determined through the optimization procedure in phase I, it was established as a constant characteristic for the specific tissue type. The following procedure was then implemented to achieve *in situ* measurement of the refractive indices and thickness for fresh samples:

(1) Hybrid system measurement: Similar to the data acquisition in step ii of phase I, the ΔD and ΔZ were acquired by the SD-OCT A-line and confocal intensity profile, respectively.
(2) Simultaneous parameter retrieval: The measured values (ΔD, ΔZ), along with the pre-determined dispersion coefficient $\eta_{\mathrm{opt}}$, were substituted into the coupled Eqs. (1)–(3). By solving this series of equations, the group refractive index ($n_{g}$), phase refractive index ($n_{p}$), and physical thickness (t) of the sample were simultaneously determined.

## 3 Results

### 3.1 Dispersion Properties

#### 3.1.1 Validation using optical window plates

To validate the accuracy and reliability of the proposed measurement strategy for dispersion coefficients, N-BK7 glass, calcium fluoride (${\mathrm{CaF}}_{2}$), and fused silica were employed as standard reference samples. First, the reliability of the measured target values ($n_{g}^{\mathrm{mea}}$ and $t^{\mathrm{mea}}$) obtained via the OPL method was verified. As summarized in Table 1, the measured group refractive indices and physical thicknesses exhibited excellent agreement with nominal values, with relative errors ranging from 0.01% to 0.09%. These precise measurements served as the ground truth for the subsequent extraction of dispersion coefficients. Subsequently, by incorporating these experimentally determined target values from the first stage, the dispersion coefficients were extracted using the hybrid system combined with the iterative optimization algorithm. As presented in Table 2, the optimized dispersion coefficients were determined to be $-0.017464\pm 0.002215\;\mu m^{-1}$ for N-BK7, $-0.008161\pm 0.004785\;\mu m^{-1}$ for ${\mathrm{CaF}}_{2}$, and $-0.015026\pm 0.001352\;\mu m^{-1}$ for fused silica. The relative errors between the measured and theoretical dispersion coefficients remained low, ranging from 0.17% (fused silica) to 5.74% (${\mathrm{CaF}}_{2}$). These results substantiate the robustness of the optimization algorithm and confirm the system’s capability to accurately characterize dispersion properties.

**Table 1:** Group refractive index and thickness measurements of optical window plates obtained via the optical path length method.

Sample	Group refractive index			Thickness (mm)
	Ref^a^	Meas	Err (%)	Meas
N-BK7	1.5243	1.5244±0.0009	0.01	1.0660±0.0007
${\mathrm{CaF}}_{2}$	1.4374	1.4387±0.0015	0.09	1.0521±0.0021
Fus Si	1.4652	1.4648±0.0006	0.02	1.0750±0.0007

a The nominal refractive indices are based on the RefractiveIndex.INFO database (https://refractiveindex.info) at a central wavelength of 860 nm.

**Table 2:** Dispersion measurements of optical window plates.

Sample	ΔD (mm)	ΔZ (mm)	Measured dispersion ($\mu m^{-1}$)	Reference dispersion ^a^ ($\mu m^{-1}$)	Err (%)
N-BK7	1.6251	0.6935	−0.017464	−0.017009	2.67
${\mathrm{CaF}}_{2}$	1.5137	0.7227	−0.008161	−0.008633	5.74
Fus Si	1.5748	0.7278	−0.015026	−0.015001	0.17

a The nominal dispersion coefficients are based on the RefractiveIndex.INFO database (https://refractiveindex.info) at a central wavelength of 860 nm.

#### 3.1.2 Dispersion of *ex vivo* porcine corneas

**Fig. 5:**
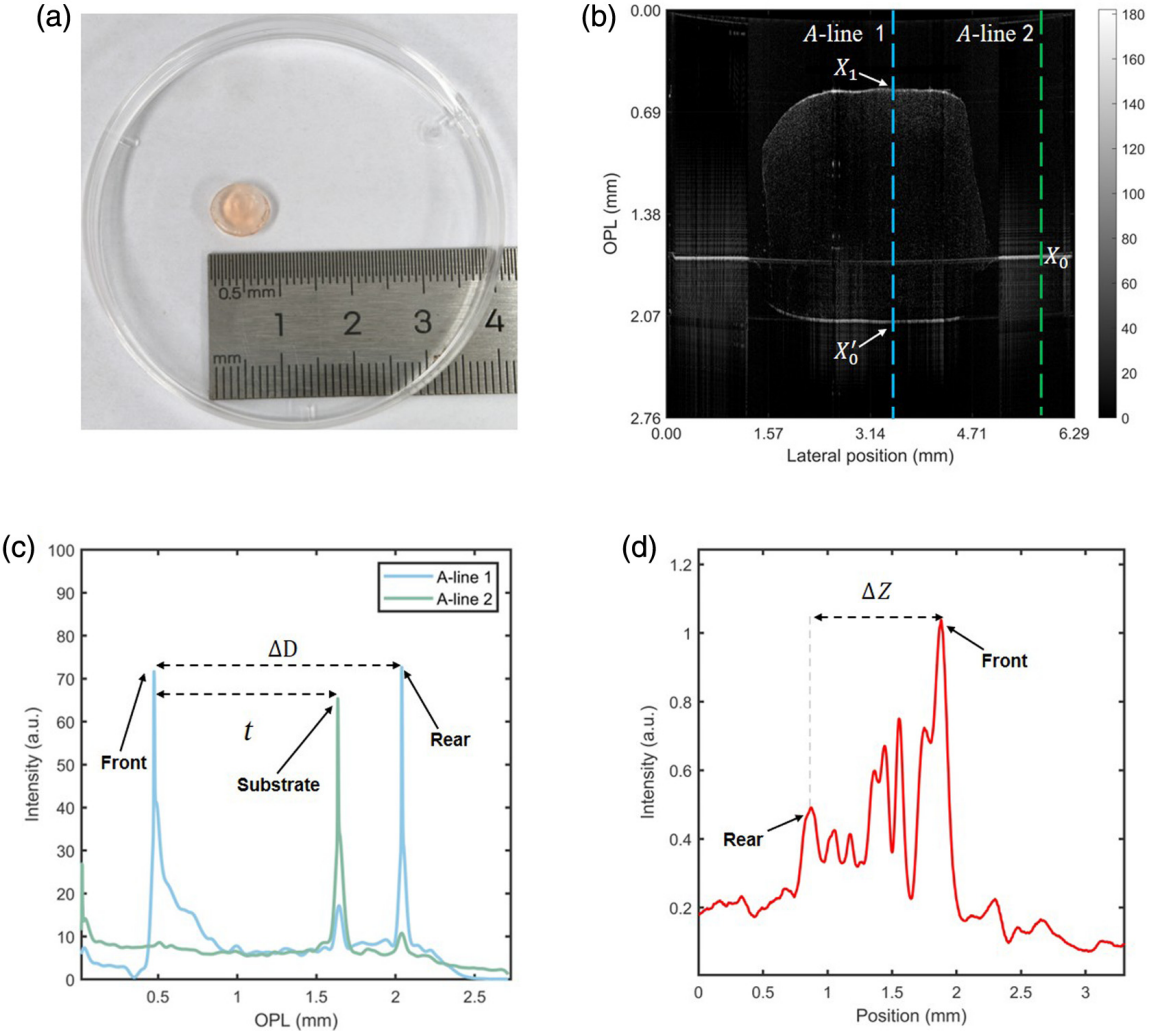
Measurement of a porcine corneal section. (a) Picture of porcine corneal section. (b) SD-OCT B-scan image. The vertical dashed lines indicate the selected A-lines. (c) Intensity profiles along the two labeled A-lines in the OCT image. (d) Confocal intensity profile. ΔD, optical path length. t, physical thickness. ΔZ, confocal distance. $X_{1}$, the OCT depth of the front surface of the sample. $X_{0}^{\prime }$, the OCT depth of the front surface of the substrate beneath the sample. $X_{0}$, the OCT depth of the front surface of the bare substrate.

Following the validation on optical window plates, the dispersion properties of five *ex vivo* porcine corneal sections were characterized. To illustrate the measurement process, sample #1 is presented as a representative example in Fig. 5(a). Figure 5(b) displays the B-scan image of the corneal section on a glass substrate. The corresponding A-line and confocal intensity profiles are plotted in Figs. 5(c) and 5(d), respectively. For sample #1, the measured optical path length, physical thickness, and confocal distance were determined to be 1.3692±0.0154  mm, 0.9910±0.0128  mm, and 0.682 mm, respectively. Table 3 summarizes the target group refractive indices ($n_{g}^{\mathrm{mea}}$) and physical thicknesses ($t^{\mathrm{mea}}$) for all five *ex vivo* porcine corneas obtained via the OPL method, alongside the corresponding system-measured parameters (ΔD and ΔZ) and the optimized dispersion coefficients. The optimized dispersion coefficients averaged $-0.010223\pm 0.002811\;\mu m^{-1}$, ranging from −0.013219 to $-0.006442\;\mu m^{-1}$.

**Table 3:** Measurements by OPL and calculated dispersion coefficients of *ex vivo* porcine cornea sections.

Sample no.	Thickness (mm)	Group refractive index	ΔD (mm)	ΔZ (mm)	Dispersion coefficients ($\mu m^{-1}$)
1	0.9910	1.3817	1.3692	0.682	−0.013219
2	1.4714	1.3732	2.0206	1.008	−0.006442
3	1.1492	1.3842	1.5907	0.827	−0.012403
4	1.0567	1.3818	1.4602	0.785	−0.008385
5	1.3531	1.3780	1.8655	0.808	−0.010667
Mean ± SD		1.3798±0.0043			−0.010223±0.002811

#### 3.1.3 Dispersion of human corneal lenticules

**Fig. 6:**
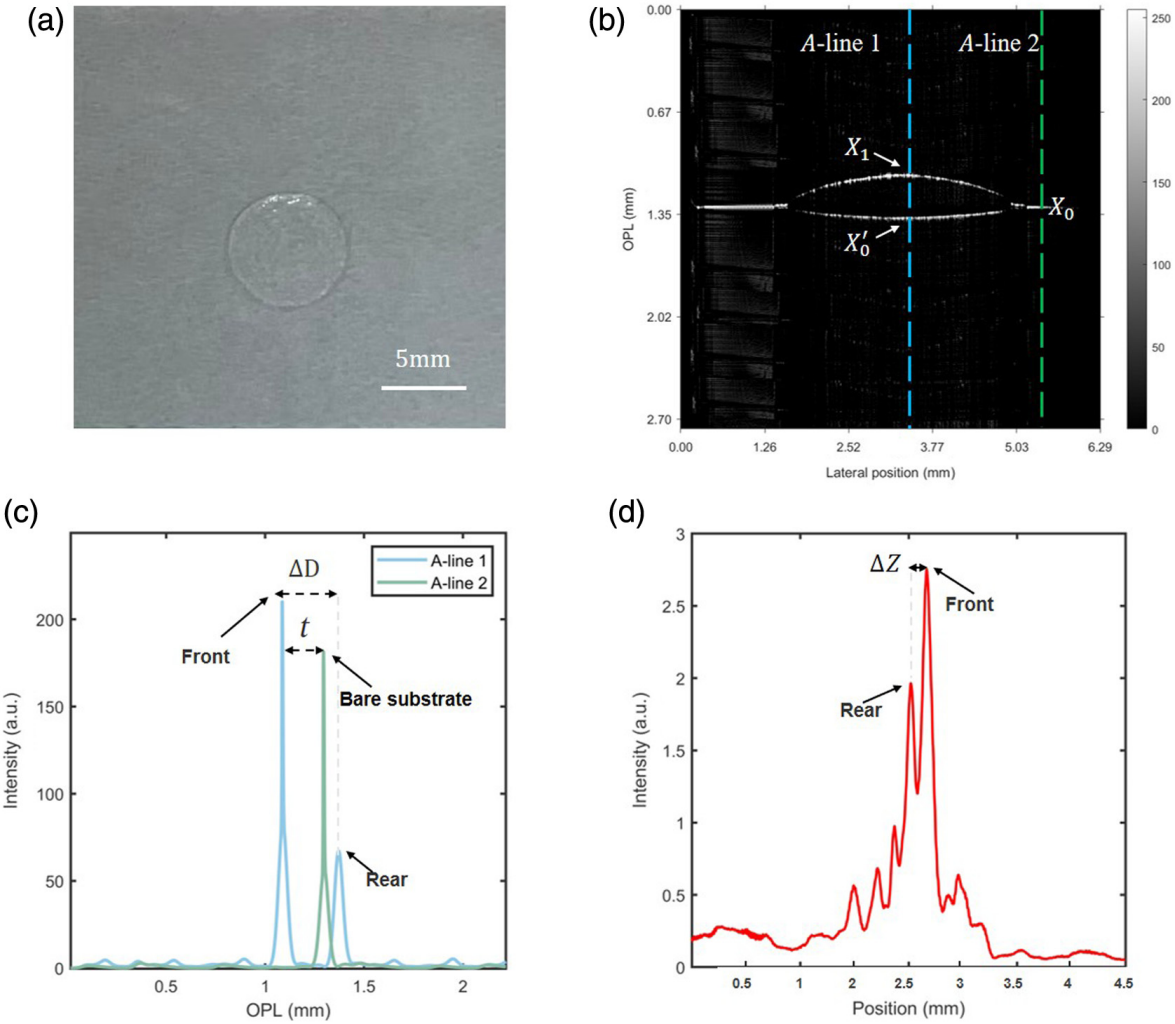
Measurement of a representative human corneal lenticule. (a) Picture of human corneal lenticule. (b) SD-OCT B-scan image. The vertical dashed lines indicate the selected A-lines. (c) Intensity profiles along the two labeled A-lines in the OCT image. (d) Confocal intensity profile. ΔD, optical path length. t, physical thickness. ΔZ, confocal distance. $X_{1}$, the OCT depth of the front surface of the sample. $X_{0}^{\prime }$, the OCT depth of the front surface of the substrate beneath the sample. $X_{0}$, the OCT depth of the front surface of the bare substrate.

Figure 6(b) displays the B-scan image of a representative human corneal lenticule on a glass substrate. As shown in Fig. 6(c), the intensity profile along the central A-line 1 exhibits two distinct peaks, which indicate the front and rear surfaces of the sample. Meanwhile, the peak in A-line 2 indicates the front surface of the bare substrate. For this representative sample, the measured optical path length and physical thickness were 0.2892±0.0330 and 0.2096±0.0023  mm, respectively. The confocal intensity profile is plotted in Fig. 6(d), and ΔZ was measured as 0.147 mm. The measurement results for all three human corneal lenticules are summarized in Table 4. The average measured group refractive index $n_{g}$ was 1.3766±0.0049. The dispersion coefficients averaged $-0.014468\pm 0.004060\;\mu m^{-1}$, ranging from −0.018128 to $-0.010100\;\mu m^{-1}$.

**Table 4:** Dispersion measurement results of the human corneal lenticule.

Sample no.	Thickness (mm)	Group refractive index	ΔD (mm)	ΔZ (mm)	Dispersion coefficients ($\mu m^{-1}$)
1	0.2096	1.3794	0.2892	0.147	−0.010100
2	0.2229	1.3794	0.3075	0.161	−0.018128
3	0.2164	1.3710	0.2967	0.157	−0.015175
Mean ± SD		1.3766±0.0048			−0.014468±0.004060

### 3.2 *In Situ* Measurement of Whole Porcine Eyes

**Fig. 7:**
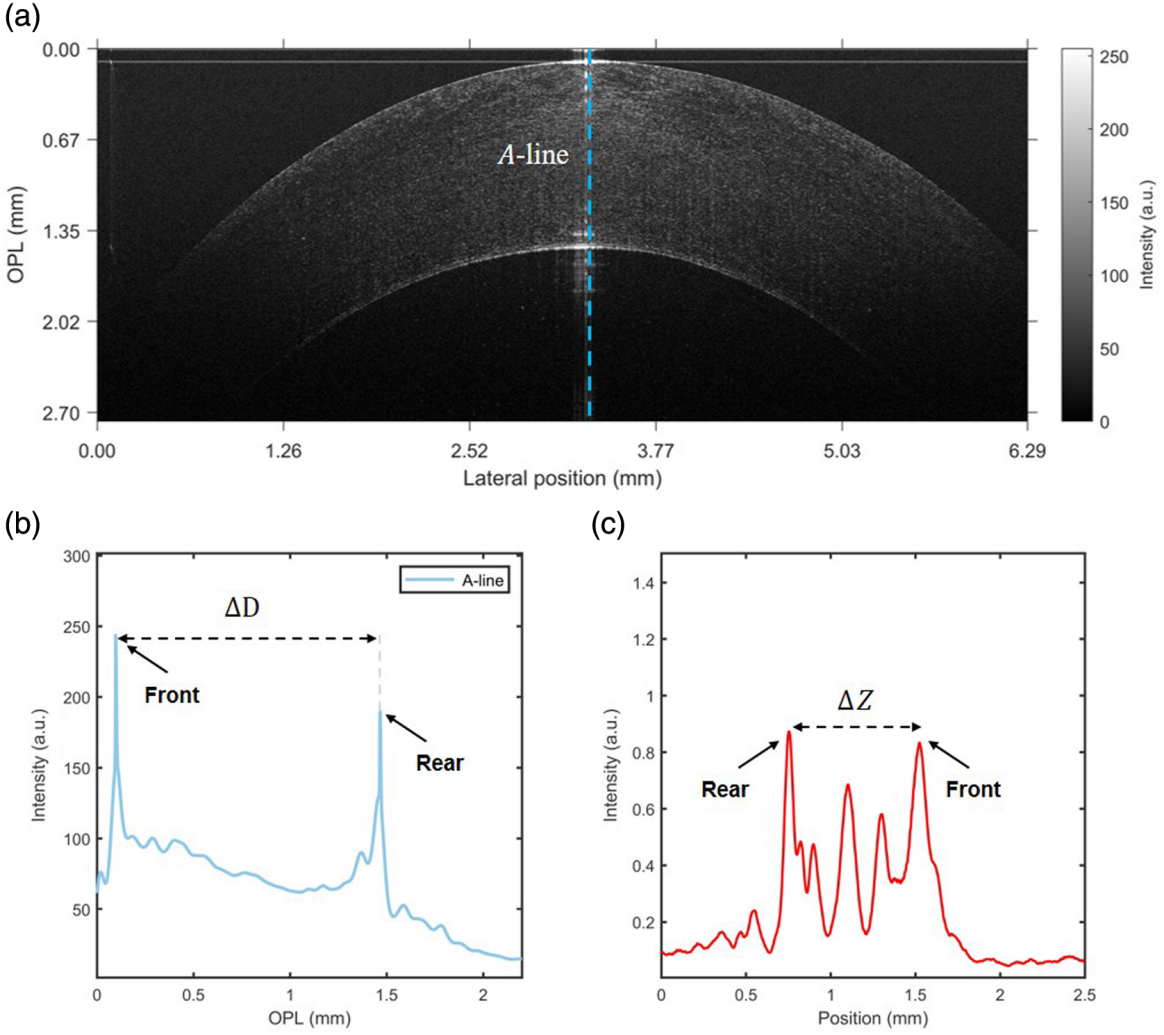
Measurement of an *in situ* porcine cornea. (a) SD-OCT B-scan image. The vertical dashed line indicates the selected A-line. (b) Intensity profile along the labeled A-line. (c) Confocal intensity profile. ΔD, *in situ* optical path length. ΔZ, *in situ* confocal distance. Front: anterior surface of the sample. Rear: posterior surface of the sample.

The average dispersion coefficient obtained from *ex vivo* corneal sections in Sec. 3.1.2 was applied as a known prior to achieve *in situ* RI decoupling for whole porcine eyes. Figure 7(a) displays the *in situ* B-scan image of a representative porcine cornea (sample #1), where the characteristic layered structure is clearly resolved. The corresponding A-line intensity profile and the confocal intensity profile are plotted in Figs. 7(b) and 7(c), respectively. For this sample, the *in situ* optical path length and confocal distance were measured as 1.3725 and 0.710 mm, respectively. Table 5 summarizes the retrieved refractive indices for all four porcine eyes. The calculated average phase refractive index ($n_{p}$) and group refractive index ($n_{g}$) were determined to be 1.3757±0.0006 and 1.3845±0.0006, respectively.

**Table 5:** *In situ* determination of refractive indices for whole porcine eyes.

Sample no.	ΔD (mm)	ΔZ (mm)	Group refractive index	Phase refractive index	Thickness (mm)
1	1.3725	0.710	1.3841	1.3753	0.9916
2	1.4305	0.739	1.3850	1.3762	1.0329
3	1.5613	0.808	1.3838	1.3750	1.1283
4	1.7272	0.892	1.3852	1.3764	1.2469
Mean ± SD			1.3845±0.0006	1.3757±0.0006	

## 4 Discussion

In this study, we demonstrated a multimodal approach integrating SD-OCT and confocal scanning to decouple the geometric and optical parameters of corneal tissue. To overcome the inherent coupling between physical thickness and refractive index in conventional interferometry, our method employs a two-step decoupling strategy by extracting the tissue dispersion coefficient as a known prior. This strategy enables the simultaneous determination of the group refractive index, phase refractive index, and physical thickness. The successful validation on optical window plates, *ex vivo* corneal tissues, and *in situ* whole eyes confirms that this scheme is robust, offering a rapid and noninvasive alternative for characterizing corneal optical properties.

Although the proposed multimodal approach successfully decouples corneal optical parameters, the current system configuration presents certain spatial-temporal limitations that merit discussion. Ideally, integrating both the SD-OCT and confocal modalities into a single coaxial scanning head would fundamentally eliminate spatial co-registration errors. However, because both modalities share the same 860 nm broadband source, utilizing a standard beam splitter in the sample arm for coaxial coupling would theoretically introduce a severe double-pass power loss (>75%). Given the highly transparent nature of the cornea and the extremely weak backscattering from its posterior boundary, such a massive insertion loss would drastically reduce the optical signal-to-noise ratio (SNR) below the acceptable threshold for robust peak detection. Consequently, a physically separated scanning architecture was adopted in this study to preserve sufficient SNR and dynamic range, albeit at the cost of requiring asynchronous data acquisition.

When using Eq. (3), our inversion model adopts an assumption that the broadband light source (860 nm center, 70 nm bandwidth) is approximated by its central wavelength. Based on our measured human corneal dispersion ($-0.0145\;\mu m^{-1}$), the maximum refractive index deviation at the extreme spectral edges (±35  nm) is only ∼0.0005. This minor deviation is practically negligible because it is largely averaged out by the symmetric spectrum and remains an order of magnitude smaller than the measured standard deviation of the group refractive index (±0.0048, Table 4). Therefore, the central wavelength approximation introduces minimal bias and provides a highly acceptable constraint for the *in situ* decoupling process.

The relative higher percentage error (5.74%) observed for ${\mathrm{CaF}}_{2}$ is inherently a mathematical artifact due to its extremely small baseline dispersion ($-0.008633\;\mu m^{-1}$) compared with the other two materials. Its absolute dispersion deviation remains on the same order of magnitude as those of the other reference materials. To evaluate the impact of prior dispersion errors on the final *in situ* decoupling outcomes, a sensitivity analysis was performed. The results shown that, when artificially introducing a 5% relative error into the *ex vivo* baseline dispersion of a porcine cornea, the output variations in both the $n_{p}$ and group refractive index $n_{g}$ were less than 0.0003, whereas the physical thickness fluctuated by merely 0.1  μm. Such infinitesimal deviations are well within the precision requirements for clinical ophthalmic applications.

The group refractive indices ($n_{g}$) measured at 860 nm for both *ex vivo* porcine corneal sections (1.3798±0.0043) and human corneal lenticules (1.3766±0.0049) demonstrate high reliability through comparative analysis. Specifically, the results for the human stroma fall strictly within the refractive index range (1.373 to 1.380) reported by Patel et al.^3^ and align with the clinical model value of 1.378 derived by de Ortueta et al.^49^ Furthermore, the measured $n_{g}$ in porcine samples is close to the previously reported refractive index values.^50^^,^^51^ This cross-species validation confirms the accuracy of the optical path length-based measurement, establishing these $n_{g}$ values as reliable target parameters for subsequent optical characterizations.

Regarding the refractive index of the *in situ* porcine cornea, Sivak and Mandelman reported a phase refractive index of 1.3739 at 650 nm.^50^ Li et al. utilized an integrated line-field OCT (center wavelength 840 nm) and Scheimpflug imaging system to calculate a mean refractive index of 1.3731.^52^ Our measured phase refractive index ($n_{p}$) of 1.3757±0.0006 at 860 nm, which falls in the near-infrared spectrum, is close to previously reported values. However, our decoupled group refractive index ($n_{g}$) of 1.3845±0.0006 exhibits a systematic discrepancy of approximately 0.01 from these citations. This discrepancy stems from the difference in physical definitions: Sivak employed Abbe refractometry, which by principle measures the phase refractive index, whereas Li explicitly assumed the group refractive index to be equivalent to the phase refractive index during their reconstruction. This numerical discrepancy underscores the necessity of our decoupling approach, whereas the deviation in $n_{g}$ highlights the inadequacy of traditional single-index models for optical path length correction in sub-micron precision interferometry. By distinguishing the difference between $n_{g}$ and $n_{p}$, we demonstrate that the derived dispersion coefficients effectively capture the intrinsic optical characteristics of the cornea tissue.

For human corneal lenticules, our measured average dispersion coefficient was $-0.014468\;\mu m^{-1}$. To validate the accuracy of this result, we introduced the classical Cauchy-equation model for ocular media proposed by Atchison et al.^47^ and calculated a theoretical baseline dispersion value of $-0.014186\;\mu m^{-1}$ at a central wavelength of 860 nm. The data comparison demonstrates that the average phase dispersion measured by our system is in high agreement with the theoretical derivation based on the classical model. This result objectively verifies the reliability of our multimodal measurement system and data inversion algorithm. Notably, this study provides the first direct experimental measurement of the dispersion coefficient specifically for the human corneal stroma. Meanwhile, physiologically, the corneal stroma exhibits a depth-dependent refractive index gradient—being denser anteriorly and looser posteriorly—and our SMILE-derived samples specifically isolate the anterior zone, thereby representing the optical properties of the corneal stromal layer. The consistency between these stromal-specific measurements and full-thickness Cauchy model predictions confirms the accuracy of our system and demonstrates its unique capability to capture individual biological heterogeneity.

Tissue morphological changes and dehydration of *ex vivo* tissues may introduce deviations in the results of optical measurements and hence the refractive indices and dispersion coefficients. Although these specific effects on *in situ* measurements were not quantitatively analyzed, they are considered negligible for two reasons. First, the *ex vivo* dispersion determination and *in situ* refractive index decoupling are two independent experimental processes. Specifically, the parameters (e.g., physical thickness, refractive index, ΔD, and ΔZ), which were acquired from the *ex vivo* tissue and may be influenced by the morphological distortions, are strictly confined to the first phase and are not directly substituted into the *in situ* calculations. Only the extracted dispersion coefficient is shared between the two models. Second, according to Eq. (3), the dispersion coefficient characterizes the wavelength-dependent trend (the derivative) of the phase refractive index curve. Although hydration causes an overall shift in the refractive index dispersion curve, its general trend remains essentially unchanged, meaning that the dispersion coefficient varies only slightly. Therefore, it is acceptable to apply dispersion coefficients obtained from *ex vivo* tissues to *in vivo* measurements under current technical conditions.

A biological limitation of the current algorithm is assuming that the cornea is a single homogeneous medium, whereas the tissue is physiologically stratified. Indeed, the confocal imaging signals in this study also captured secondary reflection peaks from these internal microstructures. However, in clinical practice, calculating critical parameters such as corneal refractive power and ocular axial length conventionally relies on a single equivalent refractive index. Practically, our algorithm extracts the actual bulk equivalent refractive index of the cornea, significantly improving measurement accuracy compared with current clinical devices that universally rely on fixed, population-averaged constants.

## 5 Conclusion

In summary, we proposed and validated an innovative multimodal method capable of simultaneously decoupling the refractive index, physical thickness, and dispersion properties of corneal tissue. By integrating SD-OCT with confocal scanning, this study pioneers the direct empirical evaluation of the dispersion coefficients for *ex vivo* porcine corneal sections and human corneal lenticules. By utilizing the derived dispersion coefficients as known priors, the system successfully achieved high-precision *in situ* measurements of physical thickness, group refractive index, and phase refractive index. This technique exhibits considerable potential for noninvasive, *in vivo* measurements of ocular tissues in clinical settings. Future work will focus on optimizing the system’s scanning speed and algorithm to further adapt to dynamic *in vivo* conditions.

## Data Availability

The data that support the findings of this article are not publicly available due to ongoing related research. However, they can be requested from the corresponding author at zhudexi@wmu.edu.cn.
